# Learning juggling by gradually increasing difficulty vs. learning the complete skill results in different learning patterns

**DOI:** 10.3389/fpsyg.2023.1284053

**Published:** 2023-11-13

**Authors:** Noga Geller, Alexandra Moringen, Jason Friedman

**Affiliations:** ^1^Department of Physical Therapy, School of Health Professions, Faculty of Medicine, Tel Aviv University, Tel Aviv, Israel; ^2^Neuroinformatics Group, Bielefeld University, Bielefeld, Germany; ^3^Institute for Data Science, Greifswald University, Greifswald, Germany; ^4^Sagol School of Neuroscience, Tel Aviv University, Tel Aviv, Israel

**Keywords:** juggling, motor learning, coordination, learning strategies, difficulty

## Abstract

Motor learning is central to sports, medicine, and other health professions as it entails learning through practice. To achieve proficiency in a complex motor task, many hours of practice are required. Therefore, finding ways to speed up the learning process is important. This study examines the impact of different training approaches on learning three-ball cascade juggling. Participants were assigned to one of two groups: practicing by gradually increasing difficulty and elements of the juggling movement (“learning in parts”) or training on the complete skill from the start (“all-at-once”). Results revealed that although the all-at-once group in the early stages of learning showed greater improvement in performance, the “learning in parts” group managed to catch up, even over a relatively short period of time. The lack of difference in performance between the groups at the end of the training session suggests that the choice of training regime (between all-at-once and learning in parts), at least in the short term, can be selected based on other factors such as the learner’s preference, practical considerations, and cognitive style.

## 1. Introduction

Motor skills are an essential component of the expertise displayed by, and required of, individuals working in medicine or other health professions, as well as the basis for many human cultural achievements, from sports to art to music. Motor learning is typically defined as a relatively permanent change in a person’s capability to perform a skill as a result of practice (Wulf, 2012). The amount of practice needed for motor skill development to achieve a high level of proficiency is dependent on the complexity of the task and can require up to thousands of hours of practice (Pritchard and Taylor, 2022). Therefore, finding effective and efficient training methods that can speed up the motor learning process are an important motivation for many researchers (Zacks and Friedman, 2020).

Motor learning is central to sport and exercise contexts and entails learning and refining skills through practice (Daumiller et al., 2021). In this experiment, three-ball cascade juggling was selected as a means to test motor learning, because it requires training and engagement of complex motor skill activities that are similar to real sports performance (Morita et al., 2016). Juggling requires simultaneous control of multiple movements, a high level of bimanual eye-hand coordination according to the visual information that is perceived, stimulating the brain areas engaged in effortful processing challenges as in open-skill sport (Berchicci et al., 2017). According to Gentile’s taxonomy (Gentile, 1990), juggling would be described as having environmental constraints of motion with intertrial variability, and to have body stability requiring manipulation.

Variability throughout the learning process has been shown to enhance learning and performance in some studies. This can be achieved by practicing different variations of the same activity or changing the task difficulty (Raviv et al., 2022). A recent study compared learning complex upper-limb movements by practicing individual movement elements or practicing the entire trajectory. The control group in the experiment learned the full complex skill, whereas two other groups learned two different movement elements of the complete skill. The results demonstrated that training on a movement element benefited the performance of the full trajectory, the two groups who learned different elements showed similar improvements in the performance of the complex motor skill, despite training on different movement elements of the same complex movement. The findings show that complex movements can be learned by practicing their movement elements (Shaikh et al., 2023). Other studies have shown mixed results regarding whether part or whole practice is more beneficial (Sattelmayer et al., 2016). In this review of medical education, they did not find an overall significant difference between the learning strategies. Another study suggested subdividing the strategy of part practice into a number of subcategories, including “increasing difficulty” (Wickens et al., 2012), which is most relevant for learning juggling. In this meta-analysis, they found that increasing difficulty can be a good strategy for learning when the increase in difficulty occurs adaptively for each participant.

For successful learning, the role of the learner’s motivation and feeling of success is significant. In a previous experiment in golf-putting, enhancing learners’ expectancies by providing a relatively “easy” performance criterion for good performance relative to a more difficult one led to more effective learning of a golf-putting task (Palmer et al., 2016). In this experiment, we compared learning in easier difficulty levels that progress to full skill difficulty with learning the task from the start at the full skill difficulty.

In our experiment, we compared acquiring three balls cascaded juggling skills between two groups - one group learned at increasing difficulty levels through practicing elements of the full movement, while the other group learned the full complex movement at one consistent difficulty level. We predict that in the early stages of training, the group that practices the whole movement will perform better, but at the end of the session, the group that practices learning in parts will overtake their performance.

## 2. Methods

### 2.1. Participants

We recruited 40 participants from the student population at the Tel Aviv University campus through flyers placed around the campus and Facebook groups. Each participant came to the lab for a single visit of approximately 1 h. The inclusion criteria were: age 18–35, and right-hand dominant. The exclusion criteria were: ADHD diagnosis or previous juggling experience.

### 2.2. Equipment

The experiment was performed with three standard juggling balls. To ensure accurate counts, the participants were filmed using a GoPro Hero 7 camera for later analysis.

### 2.3. Experiment protocol

**Figure 1:**
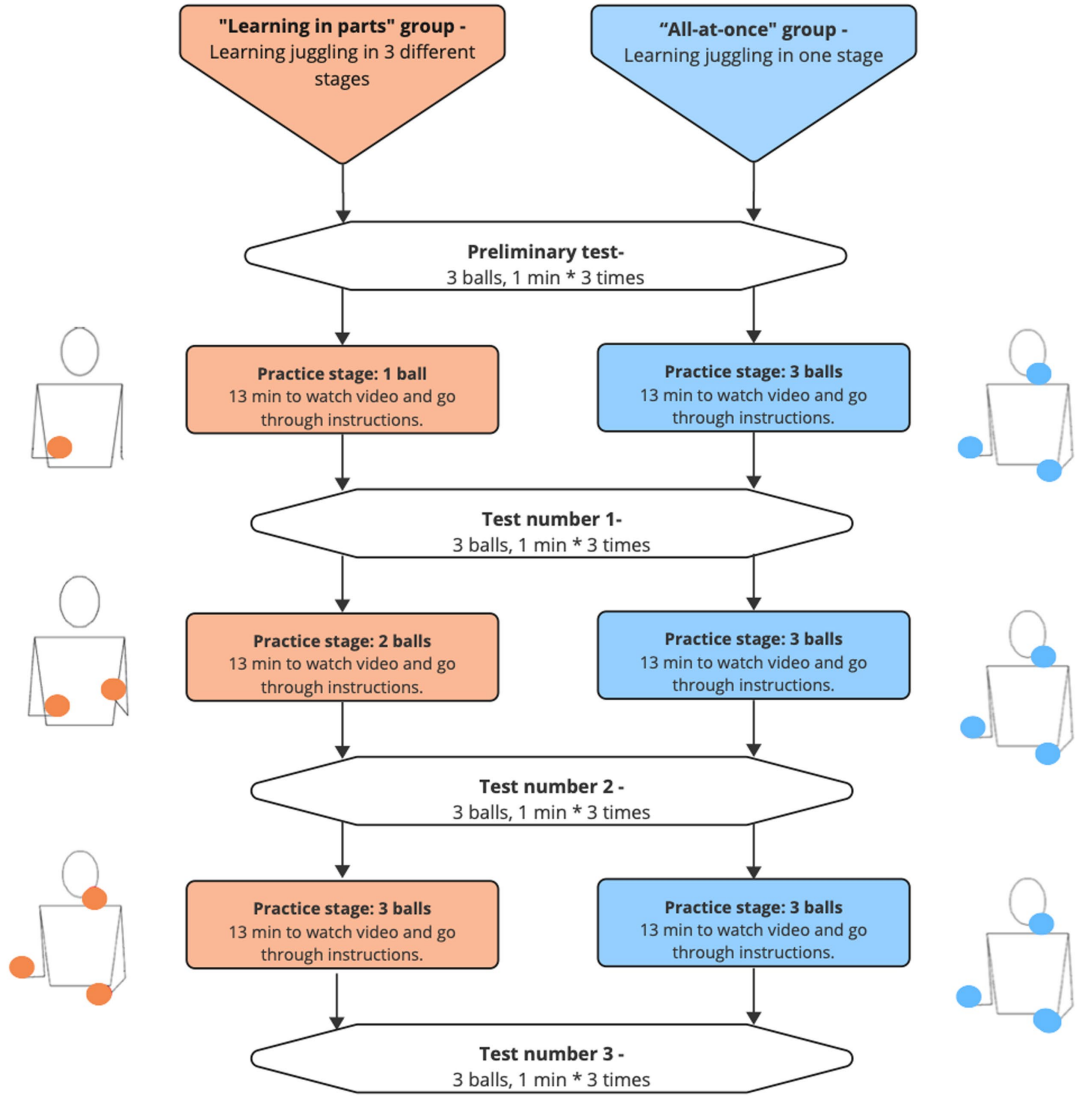
Outline of the experimental protocol. There were 20 participants in each group.

The participants were randomly assigned to one of two groups in a counter-balanced manner, which varied only in the type of training provided – learning in parts, or all-at-once. The experimental protocol is summarized in Figure 1.

Before and after each training period, the participants performed a juggling test with three balls for 1 min, repeated three times. Participants began the test while holding two balls in their right hand and one ball in their left hand. A successful three-ball juggling cascade catch was defined as follows: participants started by throwing one of the balls from the right hand diagonally across their body to the other side. As the ball reached its peak height, they threw the second ball from their left hand to the opposite side and caught the first ball. As the second ball reached its peak, they threw the third ball to the opposite side and caught the second ball. Finally, they caught the third ball. Participants earned one point for every set of three successful throws and catches, as described above, and additional points for each subsequent catch beyond the initial three. For example, if they successfully made four consecutive throws and catches, they received two points, and if they achieved five, they received three points, and so on. The total number of points for each test interval was calculated based on the cumulative number of successful catches within the one-minute juggling period. To account for potential variability in performance, participants performed the juggling test three times within each interval. The median value of the points from these three repetitions was used to determine their overall performance. The median is a statistical measure that identifies the middle value when data points are arranged in order, which can be more robust against outliers than taking the mean. The tests were recorded using a camera to confirm the counts during the tests, and the videos were solely used for this purpose.

The training consisted of three stages, each 13 min long that included 2 min of watching a video with instructions from a Youtube video “Learn to JUGGLE 3 BALLS - Beginner Tutorial” (Glenn, 2019), 1 min of going over the instructions together with the research assistant, 9.5 min of practicing juggling and 30 s rest while seated before the next test.

Details of which parts of the video were played are provided in the Supplementary material. The learning in parts group received three different sets of instructions:

1. **Juggling with one ball:** Aim for the upper corner on the opposite side of the hand that is throwing the ball and throw the ball above eye height. Keep your forearm parallel to the ground. Throw the ball vertically so that it stays close to your body.
2. **Juggling with two balls:** Start by throwing the ball in your right hand. When the first ball starts to lose height, throw the second ball. Aim with the balls for the same height above eye level. Throw the balls vertically so that they stay close to the body. To create a fixed rhythm between the balls, throw the second ball at the same time in every repetition, such that you throw the ball when the first ball starts to lose height. After enough repetitions, practice starting by throwing from the left hand.
3. **Juggling with three balls:** Initially, you should have two balls in the right hand. Start by throwing one of the balls from the right hand. Aim for the upper corner on the opposite side from the throwing hand and throw above eye height. Keep your forearm parallel to the ground. When the first ball starts to lose height, throw the ball from your left hand in the same manner. When the second ball starts to lose height, throw the third ball from your right hand. Throw the balls vertically so they stay close to each other. In order to maintain a fixed rhythm, throw the second ball at the same time each cycle so that the next ball is thrown when the previous ball starts to lose height.

The all-at-once training group received the third instruction above in each of the three training sessions. In the experiment, the participants were instructed in Hebrew, the instructions provided here are a translation. The original Hebrew instructions can be found in the Supplementary material.

All participants signed an informed consent form before starting the experiment, and the experiment received ethical approval from and was run according to the guidelines of the Tel Aviv University Institutional Review Board (IRB). The participants received payment for their participation.

### 2.4. Statistical analysis

The demographic details (age, sex) between the groups were compared using an independent samples t-test and a chi-squared test, respectively. The test scores at baseline and in the three test sessions between the two groups was compared using a non-parametric mixed-design ANOVA (F1-LD-F1 design, with the ANOVA-type statistic) with a between-subjects factor of group, and a within-subjects factor of test (baseline, and tests 1–3). F1-LD-F1 refers to a longitudinal (LD) design with 1 whole-plot factor (F1 – i.e., between-subjects - in this case group), and one subplot factor (F1 – i.e., within-subjects - in this case test) (Noguchi et al., 2012). As a measure of effect size, we used the non-parametric “measure of stochastic superiority,” which we denote as A (Vargha and Delaney, 2000; Marmolejo-Ramos et al., 2013). This measure is defined as the probability that a sample taken from one condition/group will be greater than a sample randomly taken from the other condition/group. The values range from 0.5 to 1, with 0.56 considered a small effect size, 0.64 a medium effect size, and 0.71 a large effect size (Vargha and Delaney, 2000). Non-parametric analyses were used because some of the participants had scores of 0, hence the data cannot be normally distributed. The statistical analysis was performed using R (R Core Team, 2023) with the nparLD package (Noguchi et al., 2012). *p* values for the post-hoc tests were corrected using the Holm method.

## 3. Results

Forty participants took part in the experiment divided into two groups (Learning in parts: 10 males, 10 females, mean ± SD age 25.40 ± 3.87; all-at-once: 10 males, 10 females, mean ± SD age 25.65 ± 3.92). We did not observe a significant difference in age between the groups [*t* (38) = −0.217, *p* = 0.829]. The chi-squared test did not show a significant difference in the number of male or female participants between the groups [*χ*^2^ (1) = 0.0, *p* = 1.0].

**Figure 2:**
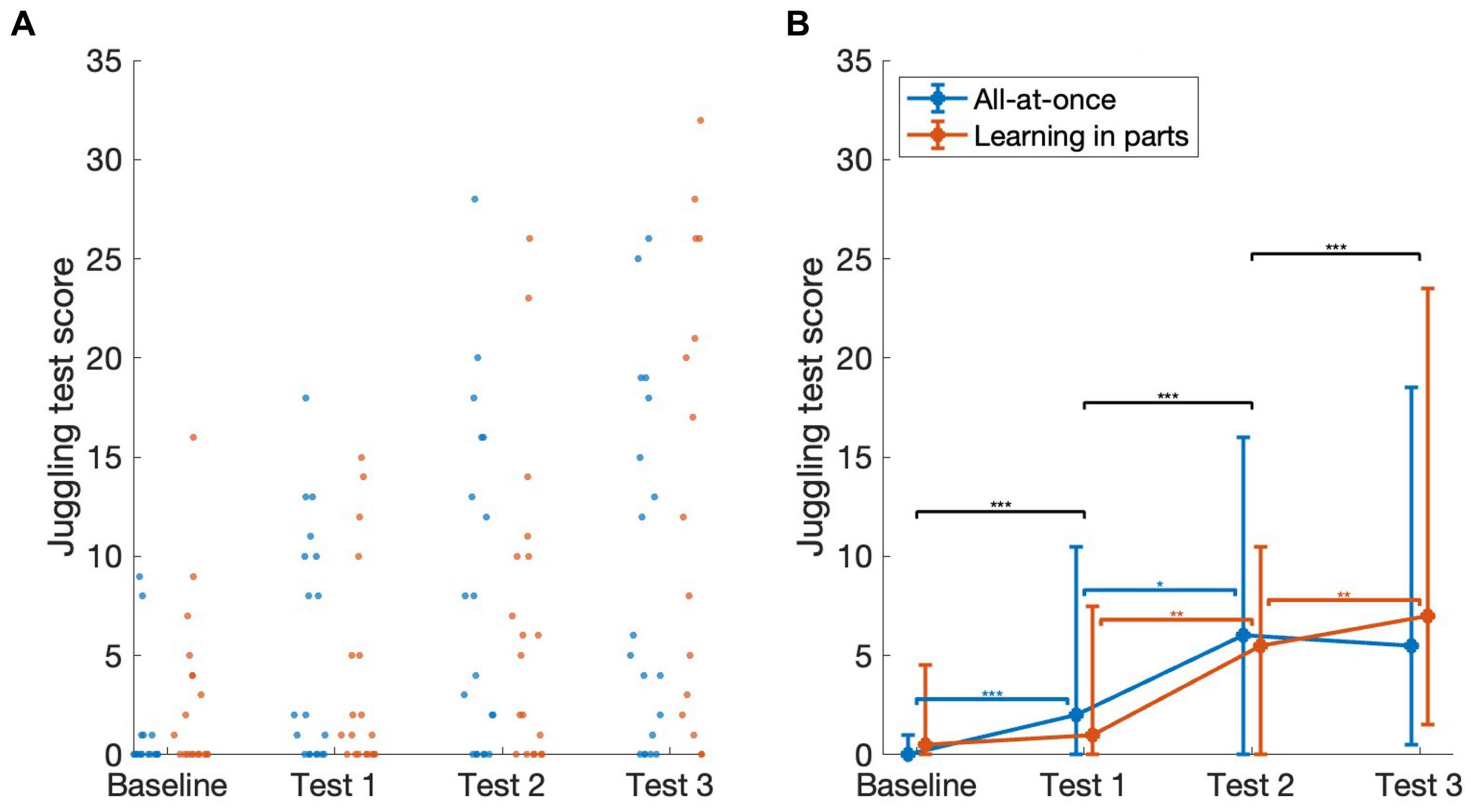
Juggling test scores of the participants in the two groups, for the baseline test and the three follow-up tests. **(A)** Juggling test scores for all subjects. For clarity, two outliers are not shown (although they were included in the statistical analysis), a graph including the outliers can be found in the Supplementary material. The data is jittered in the left–right direction to show all data points **(B)**. The medians (filled circles) and interquartile ranges (error bars) for the two groups, summarizing the data from graph **(A)**. The black horizontal bars indicate significant differences across all subjects pooled together (i.e., a main effect of test), while the blue and red bars indicate significant differences between consecutive tests for the all-at-once and learning in parts groups, respectively – the differences between the groups explain the observed interaction of test and group. *indicate *p* < 0.05; **indicates *p* < 0.01; ***indicates *p* < 0.001.

The outcomes of the tests are shown In Figure 2. A main effect was observed for test [*F* (1.652) = 38.5, *p* < 0.001]. Note that the degrees of freedom are not integers because the nparLD package uses Box-type approximations for estimating the distribution of the ANOVA-type statistics (Brunner et al., 1997). Post-hoc tests showed that the score on test 3 (median 6, IQR 1–19.25) was greater than in test 2 [median 5.5, IQR 0–13.25; *F* (1) = 15.6, *p* < 0.001, *A* = 0.74], which in turn was greater than in test 1 [median 1.5, IQR 0–10; *F* (1) = 27.4, *p* < 0.001, *A* = 0.78], which was greater than the score at baseline [median 0, IQR 0–3.25; *F* (1) = 18.3, *p* < 0.001, A = 0.69]. A main effect of group was not observed [*F* (1) = 0.028, *p* = 0.87]. An interaction of test and group was observed [*F* (1.65) = 3.64, *p* = 0.034]. Post hoc tests, after the Holm correction, showed that a significant difference between baseline and test 1 was observed only for the all-at-once group (baseline: median 0, IQR 0–1; test 1: median 2, IQR 0–10.5, *p* < 0.001, *A* = 0.78) and not for the learning in parts group (baseline: median 0.5, IQR 0–4.25; test 1: median 1, IQR 0–6.25, *p* = 0.14, *A* = 0.6). Both groups showed a significant improvement between test 1 and test 2 [all-at-once: test 2: median 6, IQR 0–16, *p* = 0.015, *A* = 0.8; learning in parts: test 2: median 5.5, IQR 0–10.25, *p* = 0.002, *A* = 0.75]. Between test 2 and test 3, a significant difference was only observed for the learning in parts group [test 3: median 7, IQR 1.75–22.25, *p* = 0.001, *A* = 0.83] and not for the all-at-once group [test 3: median 5.5, IQR 0.75–18.25, *p* = 0.114, *A* = 0.65]. We also note that by test 3, no significant difference was observed between the groups [Mann–Whitney test, *W* = 197, *p* = 0.95, *A* = 0.55].

## 4. Discussion

In this study, we examined how differences in training affect learning outcomes in a juggling task. Analysis of the results revealed that either learning to juggle three balls all-at-once, or learning by gradually increasing the difficulty and number of balls showed distinctive learning patterns, with the group learning in parts initially lagging behind but eventually catching up to the group learning all-at-once. While the group practicing the complete movement initially showed more improvement, the learning-in-parts group closed the performance gap by the end of the last test. By the end of the training session, we did not observe significant differences in performance between the groups.

In a classic experiment comparing part practice to whole practice in juggling, it was found that whole practice led to faster learning to a criterion (100 consecutive catches) than part learning (Knapp and Dixon, 1952). It should be noted that this difference was found only at *p* = 0.1, and that the Student t-test used was likely inappropriate for the data (which, based on the mean and standard deviation presented, was highly likely not to be normally distributed). Additionally, the instructions in this experiment were different from the Knapp and Dixon study, which included practice with no balls and consisted of 5 min of practice per day until they succeeded in reaching the criterion (which took up to 36 sessions). These experimental differences make it difficult to compare the two studies. In children, the choice of whole vs. part learning in juggling differed as a function of age – younger children performed better with part practice, whereas older children performed better with whole practice (Chan et al., 2015). The authors suggest that these differences may result from differences in neural maturity, information-processing ability, and motor coordination as children develop.

Consistent with the experiment’s hypothesis, the “learning in parts” group exhibited a comparatively lower improvement at the beginning of the experiment during the first test, as opposed to the all-at-once group. This outcome may be explained by the principle of Specific Adaptation to Imposed Demands (SAID), that asserts the human body adapts in very specific ways to the types of stresses we apply to them during our training, whether biomechanical or neurological. The SAID principle suggests that the more similar the training is to the desired skill, the more transferable the improvements will be to that skill (Sevier, 2000). In this experiment, the principle of specificity suggests that learning the complete 3 balls cascade juggling may lead to greater initial improvements because it closely aligns with the actual task of juggling that is tested.

However, the “learning in parts” group managed to catch up to the all-at-once group, even over a relatively short period of time. This can be explained by the different aspects of motor learning. One potential explanation is related to the concept of variability of practice. The learning in parts training group engaged in practicing different elements of the juggling movement at various difficulty levels. This variability in practice may have led to enhanced cognitive processing and adaptability (Raviv et al., 2022). However, the lack of difference between groups suggests that either strategy is effective, at least in terms of short-term training. The implication of this is that the type of training to use can be based on other considerations. Future studies may help understand also whether part training leads to more effective transfer (e.g., to four-ball juggling) than whole training (Wickens et al., 2012).

It should be noted that the results suggest that perhaps if the experiment had been conducted over a longer duration, whether through more extended practice time, or additional sessions with the participants, we might have observed an even greater improvement in the learning in parts group compared to the all-at-once group. Studies of juggling over longer time scales have shown that different participants show different learning curves (Qiao, 2021), where most of the participants showed an S-shape curve, where the rate of learning starts off relatively slowly, then accelerates, and finally decelerates before reaching a plateau. Furthermore, the motivation and feeling of success experienced by the parts training group in the training stages may have played a significant role. The concept of providing a relatively easy performance criterion, as seen in previous studies, could have encouraged a sense of accomplishment and positive reinforcement among the participants (Palmer et al., 2016).

Despite the fact that all participants selected for the experiment declared no prior knowledge of juggling and age differences were relatively small, there was a significant heterogeneity among the results of the participants, even on the baseline test. The varied results may have arisen from the fact that humans vary considerably in their ability to perform and learn new motor skills, and tasks such as juggling are highly redundant (in a kinematic sense), i.e., there are many potential ways to coordinate body movements to initially succeed at the task (Yamamoto and Tsutsui, 2021), and performance is dependent on the tempo selected (Yamamoto et al., 2018). In addition, they respond to different performance and practice conditions in varying ways (Anderson et al., 2021). Motor learning, performance and transfer are highly specific and individualized (Pacheco et al., 2019). Considerable individual differences exist even at the level of basic reaction time and all the more so for complex coordination (Anderson et al., 2021). In addition, variability in growth patterns and movement experiences likely contribute to the observed variation in initial performance levels (King et al., 2012). Thus, it seems that participants with a background in an upper-limb-involved sport like tennis or basketball may have started with a better initial performance level.

As discussed before, three balls cascade juggling is a complex motor skill that requires simultaneous control of multiple movements and a high level of bimanual eye-hand coordination (Berchicci et al., 2017). Perception and anticipation of the moving balls determines the planning of subsequent motor actions (Draganski and May, 2008). In some ways, juggling is an all or nothing task, due to the high level of motor abilities required for scoring one point. As observed in the results, many participants did not even reach a single point, even after completing all of the training.

### 4.1. Limitations

While our research exhibited some insightful findings, it is important to acknowledge and address certain limitations that might influence the results. One key limitation is the duration of our study. In this study, participants arrived for 1 hour and accumulated a total practice time (practice and tests together) of 37.5 min. The relatively short time frame allocated for the experiment might have restricted the ability to fully capture the progressive evolution of juggling proficiency within both groups, as mastery of three ball cascade juggling typically takes significantly longer - acquisition of the three-ball cascade requires three learning processes: the cognitive stage (where large demands are placed on the learner to understand the instructions and formulate strategies), the associative phase (involving proceduralizing of task strategies to enhance performance and reduce errors), and the autonomous phase (when the task becomes automatic) (Bebko et al., 2003). In another study (Morita et al., 2016), participants practiced juggling for 15 min and then underwent a total of 45 min of tests conducted at three different time points. The study concluded that participants could not fully master the juggling skill within the limited initial training and practice sessions. In other words, most subjects remained in the learning stages of juggling. Another factor that should be considered is the relatively small sample size (*N =* 40, 20 per group). A larger participant group could have potentially demonstrated clearer results with less statistical error and less effects of individual differences.

## Data availability statement

The datasets presented in this study can be found in online repositories. The names of the repository/repositories and accession number(s) can be found at: https://doi.org/10.6084/m9.figshare.24018024.

## Ethics statement

The studies involving humans were approved by the Tel Aviv University Institutional Review Board. The studies were conducted in accordance with the local legislation and institutional requirements. The participants provided their written informed consent to participate in this study.

## Author contributions

NG: Conceptualization, Investigation, Project administration, Writing – original draft, Writing – review & editing. AM: Conceptualization, Funding acquisition, Writing – review & editing. JF: Conceptualization, Formal analysis, Funding acquisition, Writing – review & editing.
