# Supplementary material for: Learning juggling by gradually increasing difficulty vs. learning the complete skill results in different learning patterns
Source: Front Psychol. 2023 Nov 13;14:1284053. doi: 10.3389/fpsyg.2023.1284053 (PMC10679398; doi:10.3389/fpsyg.2023.1284053)
Supplement: Supplementary file 1 [file Table_1.DOCX]

Supplementary Material

# Supplementary Data

The participants viewed parts of the video titled “Learn to JUGGLE 3 BALLS - Beginner Tutorial” by Taylor Glenn. The participants in the “learning in parts” group viewed from 02:36 to 03:35 in the first training session, 03:40 to 05:25 in the second training session, and 05:52 to 07:46 in the third training session. Participants in the “all-at-once” group viewed the video from 05:52 to 07:46 and watched the same video clip in all three training sessions.

The following instructions were given (in Hebrew), a translation is provided in the main text:

1. נכוון לפינה העליונה בצד הנגדי של היד שזורקת, נזרוק מעל גובה העיניים. נשמור את האמה מקבילה לקרקע. נזרוק בצורה אנכית למעלה כדי שהכדור יישאר קרוב לגוף שלנו.

2. נתחיל בלזרוק את הכדור שנמצא ביד ימין. ברגע שהכדור הראשון שזרקנו מתחיל לאבד גובה, נזרוק את הכדור השני. נכוון את הכדורים לאותו הגובה מעל גובה עיניים. נזרוק את הכדורים בצורה אנכית למעלה כדי שהם יישארו קרוב אלינו. כדי ליצור קצב אחיד בין הכדורים, נזרוק את הכדור השני בתזמון שווה כל פעם, כך שהכדור השני נזרק ברגע שהכדור הראשון מתחיל לאבד גובה. אחרי לימוד מספק, נתרגל שוב הפעם נתחיל מזריקת הכדור ביד שמאל.

3. התחלה במצב בו 2 כדורים נמצאים ביד ימין. נתחיל בלזרוק את הכדור שנמצא ביד ימין. נכוון לפינה העליונה בצד הנגדי של היד שזורקת, נזרוק מעל גובה העיניים. נשמור את האמה מקבילה לקרקע. ברגע שהכדור הראשון שזרקנו מתחיל לאבד גובה, נזרוק את הכדור שנמצא ביד שמאל באותו האופן. ברגע שהכדור השני שזרקנו מתחיל לאבד גובה, נזרוק את הכדור השלישי שנמצא ביד ימין. נזרוק את הכדורים בצורה אנכית למעלה כדי שהם יישארו קרוב אלינו. כדי ליצור קצב אחיד בין הכדורים, נזרוק את הכדור השני בתזמון שווה כל פעם, כך שהכדור השני נזרק ברגע שהכדור הראשון מתחיל לאבד גובה.

# Supplementary Figure

This is the same as Figure 2(A) from the main text but includes two outliers that were not in that figure.


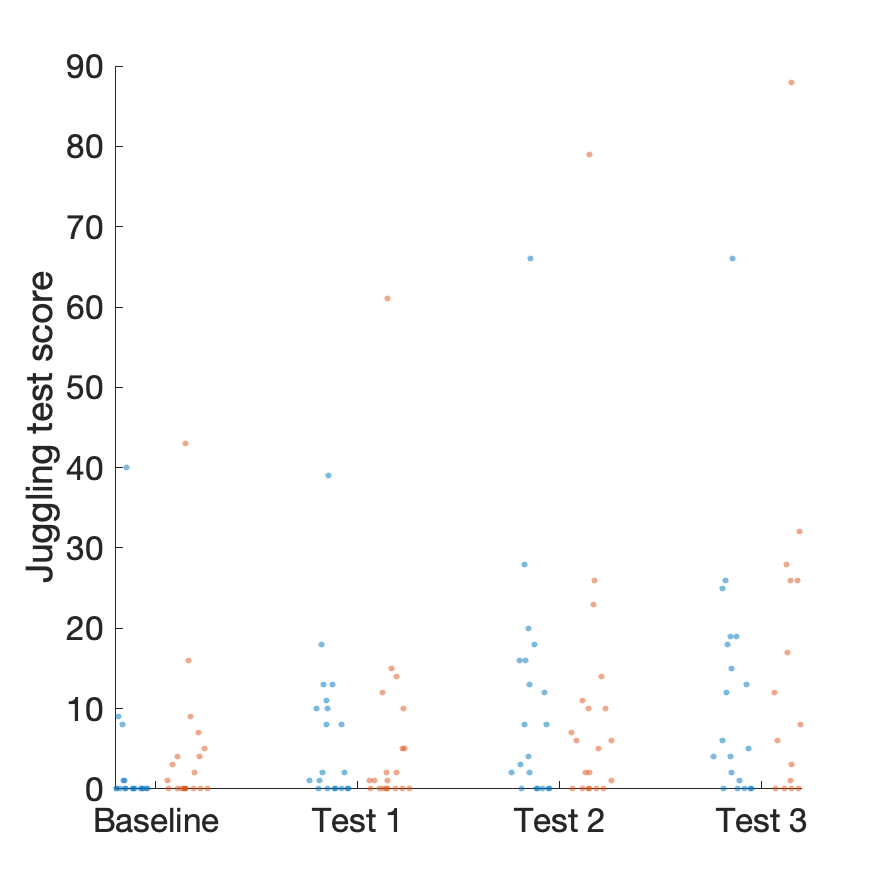


*Figure S1. Juggling test scores of the participants in the two groups, for the baseline test and the three follow-up tests. This is the same as Figure 2(A) but includes two outlier subjects.*
